# Influence of pneumatization on morphology of temporal bone-related vasculatures and their morphometric relationship with ear regions: a computed tomography study

**DOI:** 10.1038/s41598-023-29295-4

**Published:** 2023-02-03

**Authors:** Okikioluwa Stephen Aladeyelu, Samuel Oluwaseun Olojede, Sodiq Kolawole Lawal, Wonder-Boy Eumane Mbatha, Andile Lindokuhle Sibiya, Carmen Olivia Rennie

**Affiliations:** 1 Discipline of Clinical Anatomy, School of Laboratory Medicine and Medical Sciences, Nelson R. Mandela School of Medicine Campus, University of Kwazulu-Natal, Durban, South Africa; 2 Radiology Department, Inkosi Albert Luthuli Central Hospital, Durban, South Africa; 3 Lake, Smit & Partners Inc, Durban, South Africa; 4 Discipline of Otorhinolaryngology- Head and Neck Surgery, School of Clinical Medicine, Nelson R. Mandela School of Medicine Campus, University of Kwazulu-Natal, Durban, South Africa; 5 ENT Department, Inkosi Albert Luthuli Central Hospital, Durban, South Africa

**Keywords:** Anatomy, Health care, Medical research, Risk factors

## Abstract

Anatomical variations in the location and position of temporal bone-related vasculature are routinely encountered in clinical practice, contributing to clinical syndromes and complexities in ear-related and neurological surgeries. Pneumatization of the temporal bone (TB) is one of several factors that have been hypothesized to influence the variabilities and variations of these vessels. This study aimed to investigate the association between the degree of pneumatization and the morphologies of some TB-related vessels, as well as their morphometrical relationship with ear regions. Observational retrospective chart review of 496 TBs computed tomographic scans were examined. Different degrees of pneumatization were observed, with hyper-pneumatization being the most common and hypo-pneumatization being the least. Various anatomical variants of the sigmoid sinus (SS), jugular bulb (JB), and internal carotid artery (ICA) were observed. Distances of SS and JB to ear regions were observed to have significant differences (p < 0.05) in laterality. These distances increased relative to increased air cells, showing a significant association (p < 0.05). A significant association (p < 0.001) was also observed between the degree of pneumatization and variants of JB and ICA. High JB, JB dehiscence, and ICA dehiscence were significantly associated with increased pneumatization, while flat JB was significantly associated with decreasing pneumatization. However, no significant association (p = 0.070, p = 0.645) was observed between the degree of pneumatization and morphologies of SS. This study concludes that the degree of pneumatization influences only the jugular bulb variants and ICA dehiscence, as well as the distances of SS and JB to ear regions.

## Introduction

The anatomy of the temporal bone (a pair of bones located on the lateral aspect of the human skull) involves a complicated relationship between critical structures^1,2^. Its anatomical complexity poses a challenge in interpreting anatomical findings and diagnosing various pathological conditions affecting this skull region^3^.

The temporal bone (TB), though relatively small, contains the middle and inner ear structures, along with several cranial nerves and major vessels, all within a relatively small space^2^.

The location, as well as the size and shape of these vasculatures, such as sigmoid sinus (SS) and jugular bulb (JB) (a connection between the SS and the internal jugular vein), is highly variable, including a significant difference in laterality^2,4–6^. The pneumatization of the TB has been reported to be responsible for these variabilities^7,8^. This was also indicated in the study of Singh et al*.*^9^, highlighting how pneumatization of the TB influences the morphology of the SS.

Anatomical variations of these vasculatures and their anomalies, such as protrusive SS, dehiscence SS, high or high riding JB, and JB dehiscence, are not rare in the TB^10,11^. For instance, high JB has been seen to occur in 6–20% of the population^11^. Other reports found variations in the incidence of high JB, with some citing as low as 3.5% and others reporting high incidences up to 65%^11–14^.

These variations of the SS and JB are frequently encountered in clinical practice, affecting the middle and inner ear functions and causing tinnitus, vertigo, and/or hearing loss^15^. The variations also impact the complexity of neurotological surgeries and are a vital pitfall encountered in TB and ear-related surgeries^2,11,16^. For example, injuries to these vessels due to surgical trauma have been mostly observed after mastoidectomy procedures^11^. Their significance, even in relatively minor otological surgery, was highlighted in a case of a myringotomy in a 10-year old boy with acute otitis media^16^. The myringotomy of a dark-blue tympanic membrane led to massive bleeding and subsequent death of the child following thrombosis of an iatrogenically injured dominant SS^16^.

Acute otitis media requiring, minor otological surgeries and TB-related fractures resulting in surgical intervention are not rare in South Africa, sub-Saharan Africa, and other regions of the world^17–22^. Most TB and ear-related surgeries, depending on the approach, require a good knowledge of the degree of TB pneumatization and the relationship between SS, JB, the external acoustic canal (EAC), the middle ear (ME), and the internal acoustic canal (IAC) in order to avoid intraoperative complication^2^. For instance, the SS is one of the most familiar landmarks used during trans-mastoid and posterolateral skull base approaches^23,24^. It can also be used as a reference structure when evaluating the degree of mastoid pneumatization^25^.

Since the studies of Ichijo et al*.*^23^ and Dai et al*.*^26^ that hypothesized possible influences of mastoid pneumatization on the position of SS, observable and measurable data concerning the influence of TB pneumatization on the morphology of TB-related vessels and their morphometric relationship with ear-related structures/regions remain scarce. Hence, this study sets out to investigate any possible influence TB pneumatization may have on the morphology and morphometry of its related vasculature by measuring the shortest distances between selected vasculature (such as SS and JB) and EAC, ME, and IAC, evaluating their vascular variants, and analyzing for any possible association with the degree of pneumatization of the TB.

## Methods

### Study population

The present study was an observation and retrospective review of 496 TBs computed tomography (CT) images of 248 head and neck/brain CTs of patients retrieved from the Picture Archiving and Communication Systems (PACS) of public hospitals in Durban and Pietermaritzburg, Kwazulu-Natal, South Africa. The three population groups identified in the study were South African Blacks, South African Indians, and South African Whites.

### Inclusion criteria

These include high-resolution multi-detector CT images acquired with 0.6 mm collimation, images without observable signs of abnormal pathological processes in the TB, and absence.

### Scanning protocol

The CT images were acquired using Multi-Detector row Computed Tomography (MDCT) Scanners (Lightspeed CT, GE Healthcare Medical System, Milwaukee, Wisconsin, USA and SOMATOM Definition Flash CT Scanner, Siemens Healthineers, Forcheim, Germany, both of 64 and 128 slice configuration respectively). The scans were performed in a craniocaudal topographical direction using 140 kV, modulated mAs ranging between 280 and 400 mA (beam collimation 64 × 0.625; rotation 0.5 s) with 30% dose reduction and ASIR-V application in a bony algorithm with a window width of > 3000 hU and a window level of 500 hU. The axial view was reconstructed parallel to the orbito-meatal line using slice thickness of 0.625 mm, detector coverage of 20 mm, and a PITCH of 0.5. Voxel size of images is isotropic.

### Morphological and morphometrical analyses

Using digital imaging and communication in medicine (DICOM), TB CT images retrieved from the PACS of public hospitals selected for this study were transferred to a Workstation, and all morphological and morphometrical analyses were carried out using IntelliSpace Portal (ISP) Version 11.1 (Philips Image and Information Management software, Nederland). Prior to the morphologic and morphometric analyses of images selected for this study, a pilot study was initially conducted on 20 TB CT images in other to ascertain specific landmarks for analysis of vascular variants, degree of pneumatization, and distant measurements. Also, 50 randomly selected temporal CT images (both left and right) were analyzed by a first, second, and third observer (a Clinical Anatomist, a Radiographer, and a Specialist Radiologist- Head and Neck, respectively) for inter-observer reliability testing. This was done to achieve the accuracy and repeatability of measurements.

### Morphometric analysis of SS and JB from the ear region (EAC, ME & IAC)

**Figure 1:**
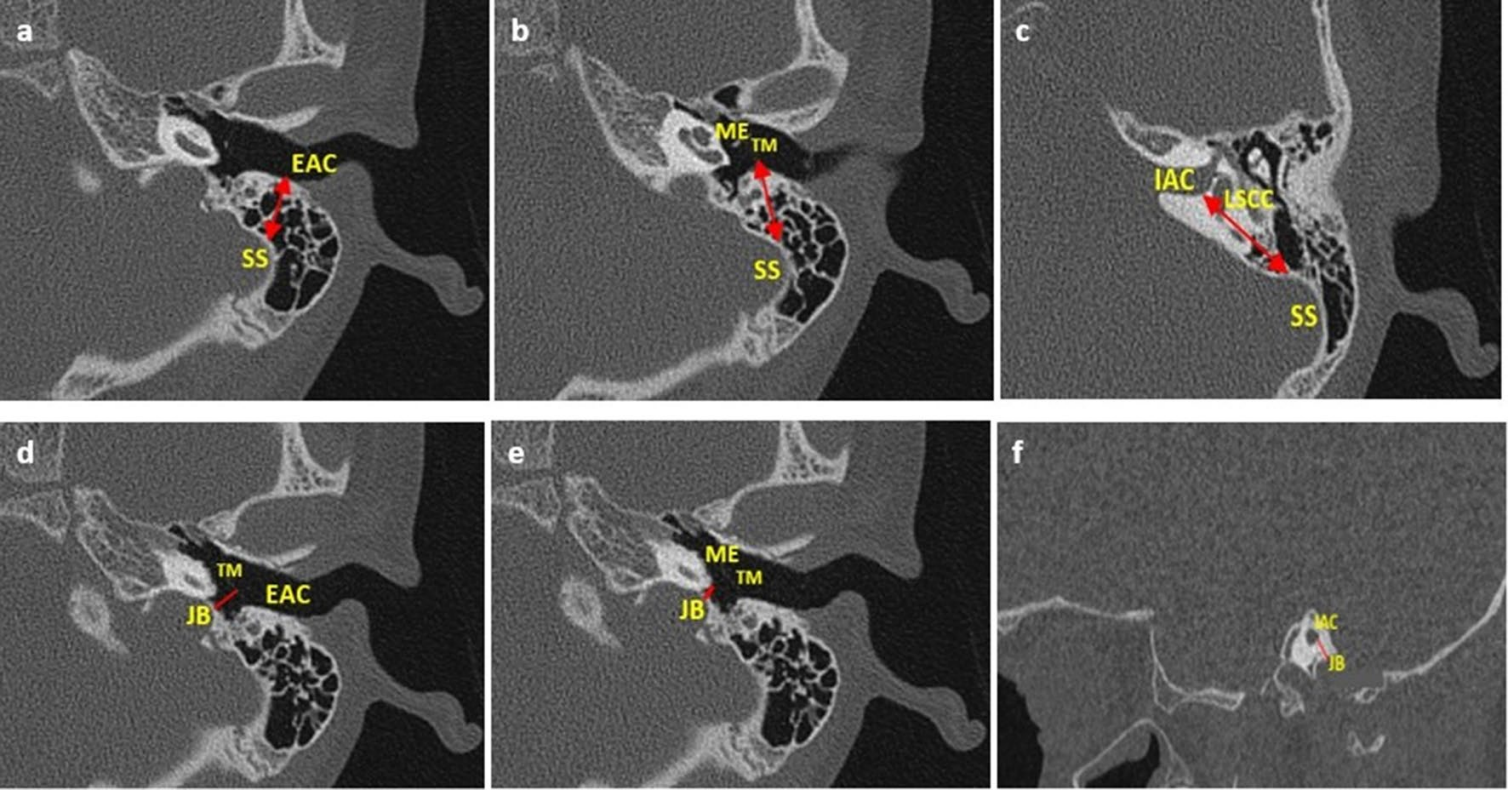
Diagrammatic representation of all measurements. *SS* sigmoid sinus, *JB* jugular bulb, *EAC* internal acoustic canal, *ME* middle-ear, *IAC* internal acoustic canal, *TM* tympanic membrane, *LSCC* lateral semicircular canal*.* (**a**) SS – EAC (on the axial plane, a double-tipped red arrow from the apex of SS to the posterior wall of EAC); (**b**) SS – ME (on the axial plane, a double-tipped red arrow from the anterior edge of SS to the tympanic membrane); (**c**) SS – IAC (on the axial plane, a double-tipped red arrow from the anterior edge of SS to the posterior wall of IAC); (**d**) JB – EAC; (on the axial plane, a red line from JB to the tympanic border); (**e**) JB – ME (on the axial plane, a red line from JB to the sigmoid plate); (**f**) JB – IAC (on the sagittal plane a red line from the dome of JB to the inner ear).

The shortest distances between the selected TB-related vessels (SS and JB) and ear regions (EAC, ME & IAC) were achieved as described in Fig. 1. The points of measurement were standardized based on surgical relevance. Axial images at the level or about the level of the lateral semi-circular canal were used (visible landmarks of ear regions are best identified around this level). SS to EAC- from the apex of SS to the posterior wall of EAC; SS to ME- from the anterior wall of SS to the tympanic membrane (TM); SS to IAC- from the anterior edge of SS to the posterior of IAC; JB to EAC- from the apex of the bulb to tympanic border; JB to ME- from the apex of the bulb to the sigmoid plate; JB to IAC from the dome of the bulb to inner ear.

### Morphological analysis of vascular variants

**Figure 2:**
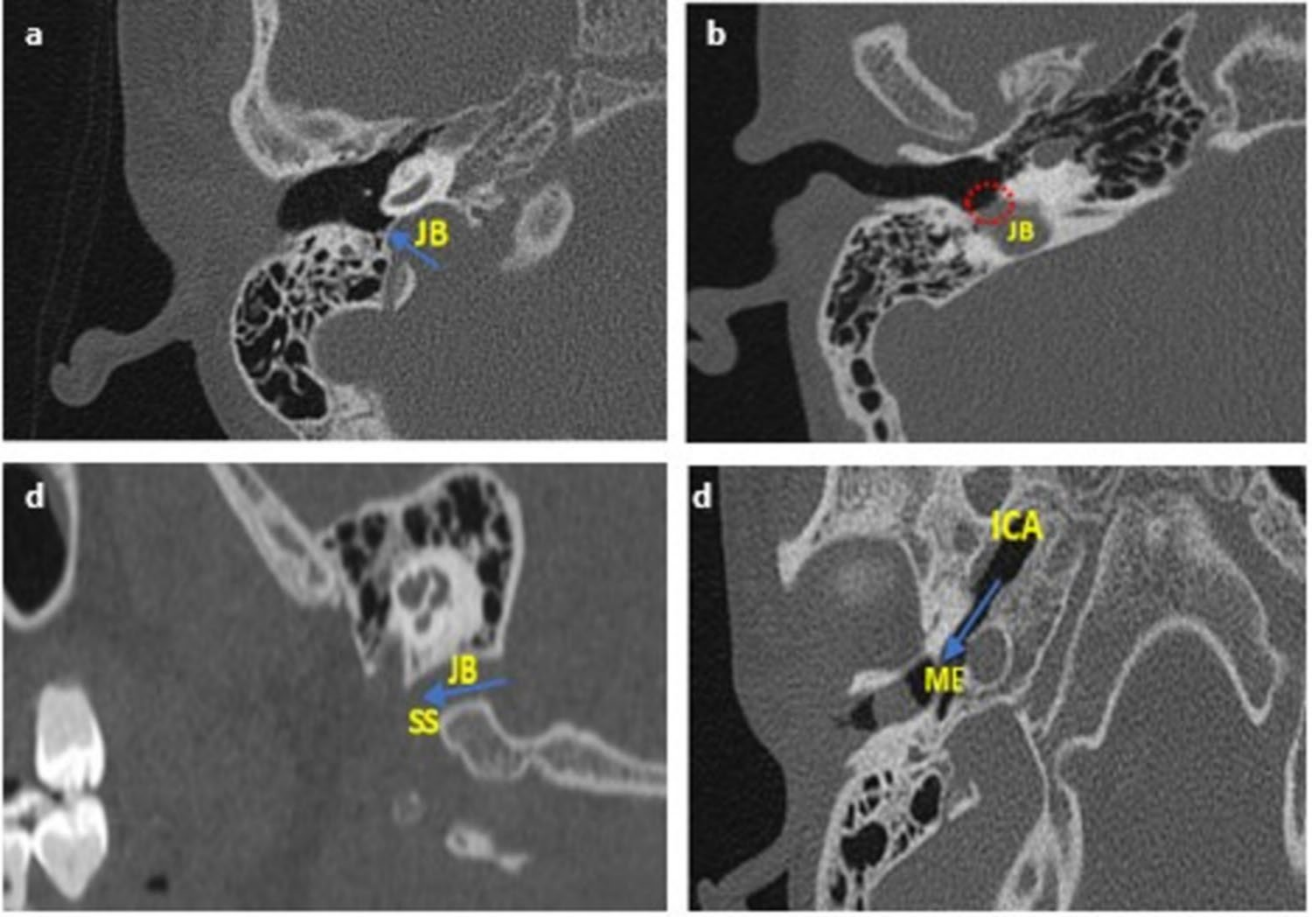
Diagrammatic representation of all vascular variants on the axial and sagittal plane. *JB* jugular bulb, *ICA* internal carotid artery, *ME* middle-ear, *SS* sigmoid sinus. (**a**) HJB- blue arrow showing the bulb rising to the sigmoid plate; (**b**) JBD- red dotted circle showing absence of sigmoid plate due to descent of JB into the middle ear; (**c**) FJB- absence of the dome (rising bulb), blue arrow showing SS continue into the internal jugular vein; and (**d**) ICA-Deh: blue arrow showing focal dehiscence of the carotid canal into the middle ear.

**Figure 3:**
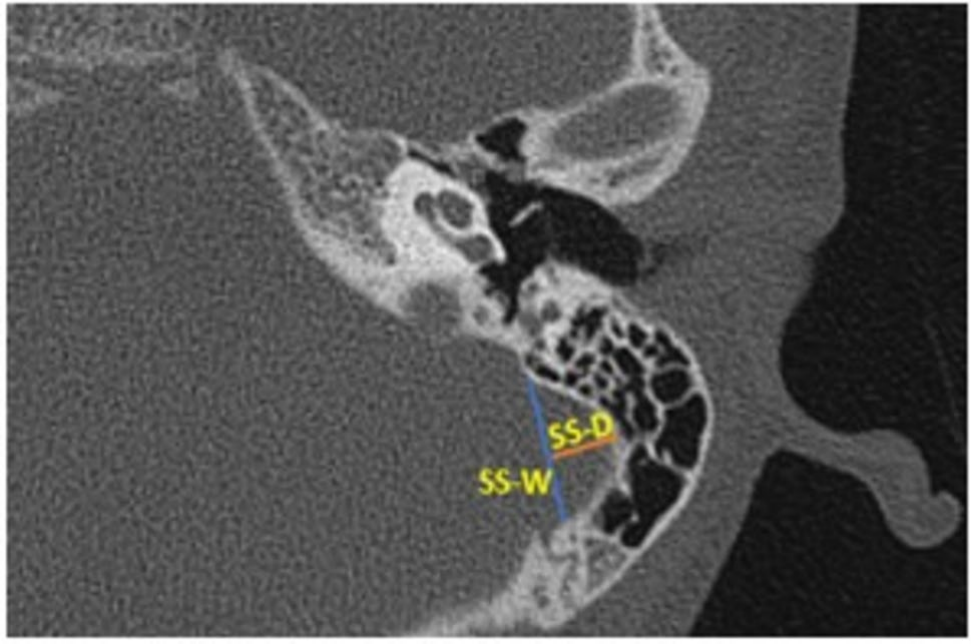
Diagram showing the measurements of sigmoid sinus width and sigmoid sinus diameter. *SS* sigmoid sinus, *W* width, *D* diameter. SS-W (blue line) and SS-D (orange line).

The variants of the jugular bulb analyzed in the study are as follows: high jugular bulb (when the bulb's dome rises close to the tympanic membrane’s annulus), jugular bulb dehiscence (when HJB extends into the middle-ear cavity due to dehiscent sigmoid plate), and flat jugular bulb (no bulb or absence of the dome) (Fig. 2a–c, respectively). The internal carotid artery was analyzed for ICA dehiscence into the middle ear (Fig. 2d). Finally, for the sigmoid sinus, the following morphologies were analyzed based on shape: Half-moon type (SS-Diameter = **½**SS-Width), Protrusive type (SS-Diameter > **½**SS-Width), and Saucer type (SS-Diameter < **½**SS-Width). SS-Diameter and SS-Width were achieved as described in Fig. 3.

### Degree of pneumatization

**Figure 4:**
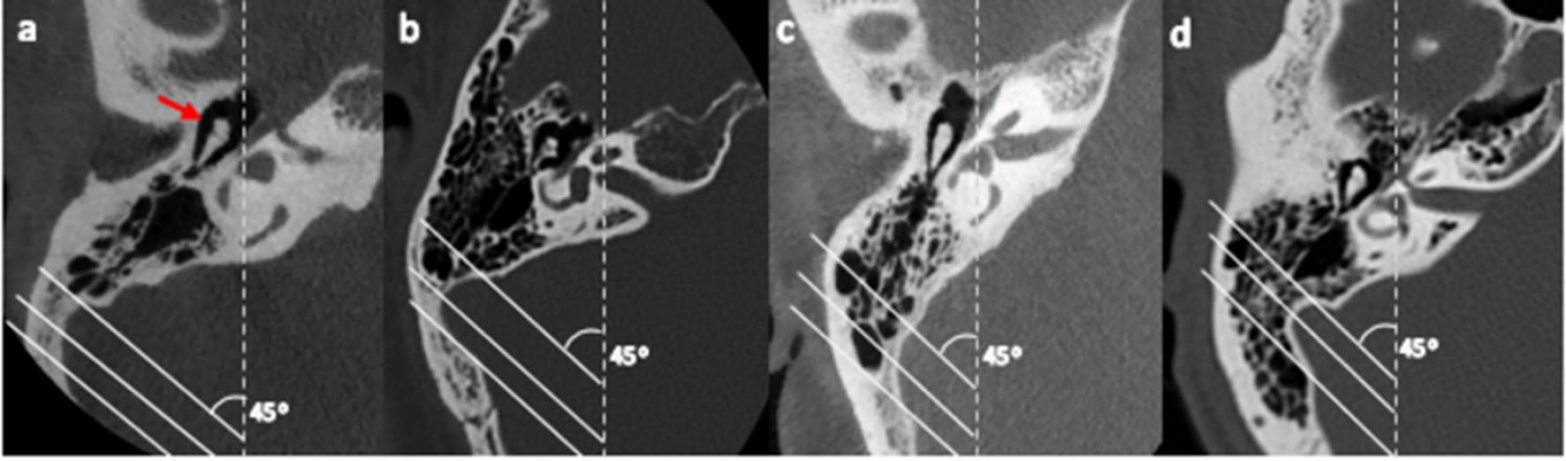
Degrees of pneumatization by evaluating air cells around the sigmoid sinus. Axial section where the malleoincudal complex (red arrow) appeared as an ice-cream-cone shape. Three parallel lines were drawn angled at 45˚ to the anteroposterior axis (dotted line). (**a**) Hypo-pneumatization: pneumatization remains anteromedial to the anterior line (passing the most anterior point). (**b**) Moderate pneumatization: pneumatization between the anterior and middle lines (passing the most lateral point). (**c**) Good-pneumatization: pneumatization between the middle and posterior lines (passing the most posterior point). (**d**) Hyper-pneumatization: pneumatization extends posterolaterally beyond the posterior line.

Following the proposed classification system by Han et al*.*^25^, the degree of TB pneumatization was analyzed by evaluating the air cells around the sigmoid sinus on an axial CT image. This involved the application of three parallel lines in the anterolateral direction angled at 45° on the axial CT section, where the malleoincudal complex appears as an ice-cream-cone shape. These lines were applied at positions in which each crossed the most anterior point of the sigmoid sinus at the junction with the petrous bone, the most lateral aspect along the transverse plane of the sigmoid groove, and the most common posterior point of the sigmoid sinus, respectively (Fig. 3). Hence, the degree of pneumatization was classified into four groups: Hypo-pneumatization- pneumatization that extends to the line drawn at the most anterior aspect of the SS (Fig. 4a); Moderate pneumatization- pneumatization that extends to the space between arbitrary lines drawn at the most anterior point and most lateral aspect of the sigmoid sinus (Fig. 4b); Good pneumatization- Pneumatization that extends to the space between the lines drawn at the most lateral region and the most posterior point of the SS (Fig. 4c) and; Hyper-pneumatization- pneumatization that extends postero-laterally beyond the line drawn at the posterior point of the sigmoid sinus (with possible extension into the petrous- extensive pneumatization) (Fig. 4d).

### Statistical analysis

The statistical data analysis was conducted in the R Statistical computing software of the R Core Team, 2020, version 3.6.3. The results were presented in the form of descriptive and inferential statistics. The numeric measurements were non-normal and summarized in median and interquartile ranges. The categorical variables were presented in numbers and percentages. The median differences between two groups were assessed using the Wilcoxon test. The median differences across at least three levels of a categorical variable were assessed with the aid of Kruskal Wallis. In the case of significant median difference, post-hoc tests were conducted using Dunn test. Chi-Square tests were applied to determine the association between categorical variables, and when the distribution of the cross-tabulations contained an expected value of less than five, a Fisher’s exact test was applied. All the inferential statistical analysis tests were conducted at 5% significance levels.

### Ethical approval

The study was approved by the Biomedical Research Ethics Committee of the University of KwaZulu-Natal (Protocol Ref. No.: BREC/00002263/2020) and the National Health Research Committee of the KwaZulu-Natal Department of Health (NHRD Ref.: KZ_202102_026). All methods were carried out in accordance with the University of KwaZulu-Natal standard-approved guidelines and regulations, as well as all experimental protocols in accordance with the declaration of Helsinki.

## Results

**Figure 5:**
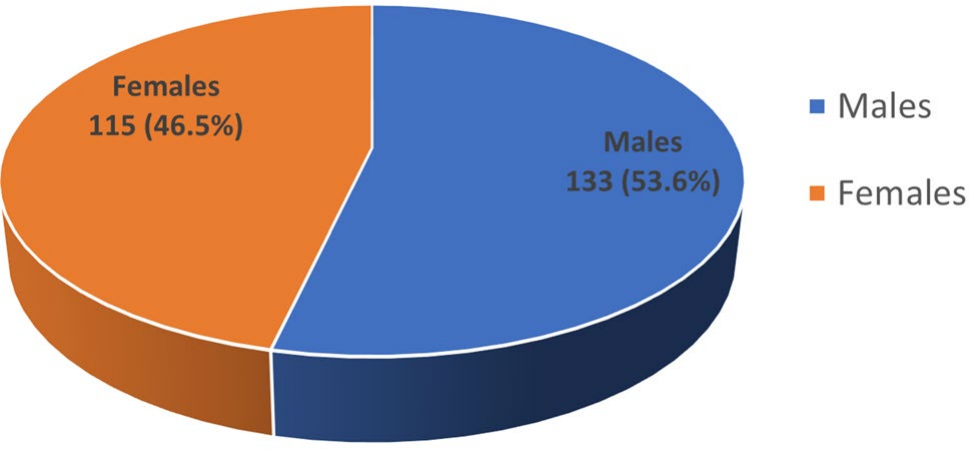
Pie chart of sex distribution of patients.

All continuous and categorical data were presented as the median and interquartile range (IQR) and percentage (%). A total of 248 patients with 496 HRCT TB scans (right and left sides) were included in this study. The median age of the patients was 13.0 years (interquartile range, 8.0–23.0). Of the patients, 115 (46.4.5%) were females, and 133 (53.6%) were males (Fig. 5).

For inter-observer reliability testing for all linear measurements, the intraclass correlation coefficient was 92% for SS-EAC length, 91% for SS-ME length, 92% for SS-IAC length, 90% for JB-EAC length, 85% for JB-ME length, 87% for JB-IAC length, 92% for SS-diameter, and 93% for SS-Width.

### Overall degree of pneumatization of the temporal bone

The degree of pneumatization was achieved by evaluating air cells around the sigmoid sinus, according to Han et al.^26^*.* The degree of pneumatization was classified into four groups; hypo-, moderate-, good- and hyper- pneumatization. The most common degree of pneumatization is hyper-pneumatization (140, 37.9%), where pneumatization extends beyond the arbitrary line drawn at the most posterior point of the SS. However, females were more likely to have hyper-pneumatization (73, 41.2%), while males were more likely to have hypo-pneumatization (31, 16.1%). Also, hypo-pneumatization was observed to have a higher incidence on the left, while moderate and good pneumatization had higher incidences on the right. In most patients, the degree of pneumatization was observed to be the same on both sides of the TB, with a significant association at p < 0.05. The results are detailed in Table 1.

**Table 1:** Overall degree of temporal bone pneumatization, including sex and laterality.

Degree of pneumatization	Female			Male			Overall		
	Left *f (%)*	Right *f (%)*	Total *f (%)*	Left *f (%)*	Right *f (%)*	Total *f (%)*	Left *f (%)*	Right *f (%)*	Total *f (%)*
Hypo	14 (15.7)	11 (12.5)	25 (14.1)	14 (14.3)	17 (18.1)	31 (16.1)	28 (15.0)	28 (15.4)	56 (15.2)
Moderate	17 (19.1)	19 (21.6)	36 (20.3)	24 (24.5)	25 (26.6)	49 (25.5)	41 (21.9)	44 (24.2)	85 (23.0)
Good	19 (21.3)	24 (27.3)	43 (24.3)	24 (24.5)	21 (22.3)	45 (23.4)	43 (23.0)	45 (24.7)	88 (23.8)
Hyper	39 (43.8)	34 (38.6)	73 (41.2)	36 (36.7)	31 (33.0)	67 (34.9)	75 (40.1)	65 (35.7)	140 (37.9)

*f* frequency; *%* percentage.

### Variations in the morphometric relationship between SS and JB and the ear regions (EAC, ME & IAC.)

The average distances of SS to EAC, ME, and IAC and JB to EAC, ME, and IAC were observed to be greater on the left. According to laterality, the Wilcoxon rank test showed a significant difference in average distances of SS-EAC, SS-ME, SS-IAC, JB-ME, and JB-IAC (p < 0.01) except for JB-EAC, which showed no significant difference (p = 0.55) (Table 2). For sex, there was no significant difference observed in distances of SS-ME (Right, p = 0.718; Left, p = 0.679), SS-ME (Right, p = 0.608; Left, p = 0.776), SS-IAC (Right, p = 0.618; Left, p = 0.086), JB-EAC (Right, p = 0.389; Left, p = 0.859), JB-ME (Right, p = 0.914; Left, p = 0.749), and JB-IAC on the left (p = 0.292). However, a significant difference (p < 0.05) was observed in the average distance of JB-IAC on the right, with the females having a distance of 7.56 (6.11–10.1) mm and the males having a distance of 6.78 (4.97–8.17) mm (Table 3). There was no significant difference observed with age for all measurements.

**Table 2:** Average distances of the sigmoid sinus and jugular bulb from selected ear region (external acoustic canal, middle-ear & internal acoustic canal) according to side differences.

	SS-EAC median (IQR) *mm*	SS-ME median (IQR) *mm*	SS-IAC median (IQR) *mm*	JB-EAC median (IQR) *mm*	JB-ME median (IQR) *mm*	JB-IAC median (IQR) *mm*
Right (a)	15.5 (13.8–17.7)**	12.6 10.8–14.9)***	19.0 (16.4–21.8)***	13.0 (10.2–16.0)	7.10 (3.82–10.5)***	7.11 (5.57–9.08)*
Left (b)	15.9 (13.7–18.0)**	13.2 (11.4–14.4)***	20.1 (18.0–21.9)***	13.1 (9.95–15.9)	7.70 (4.00–10.2)***	7.49 (6.03–8.85)*
a vs. b (sig. diff) *p-value*	0.006	0.0027	0.002	0.55	0.0045	0.041

*mm* millimeters, *IQR* Interquartile range, *SS* sigmoid sinus, *JB* jugular bulb, *EAC* internal acoustic canal, *ME* middle-ear, *IAC* internal acoustic canal.

Wilcoxon rank test showing *Significant different with p < 0.05; **Significant different with p < 0.01; ***Significant different with p < 0.005.

**Table 3:** Morphometric analysis (distance) of the sigmoid sinus and jugular bulb from ear region (external acoustic canal, middle-ear & internal acoustic canal) according to sex.

	Right			Left		
	Female (1) (N = 115)	Male (2) (N = 133)	(1) vs (2)	Female (3) (N = 115)	Male (4) (N = 133)	(3) vs (4)
SS–EAC median (IQR) *mm*	16.0 (13.9–17.6)	16.0 (13.8–17.9)	p = 0.718	16.00 (13.6–17.9)	15.7 (14.0–18.1)	p = 0.679
SS–ME median (IQR) *mm*	12.9 (10.6–14.7)	13.0 (10.8–15.0)	p = 0.609	12.9 (11.4–14.4)	12.5 (11.4–14.5)	p = 0.776
SS–IAC median (IQR) *mm*	19.7 (16.4–21.7)	19.1 (16.0–22.2)	p = 0.618	20.10 (19.2–22.2)	20.10 (17.1–21.5)	p = 0.086
JB–EAC median (IQR) *mm*	13.1 (10.5–16.0	12.0 (9.90–16.1)	p = 0.389	13.3 (10.2–15.1)	12.7 (9.91–16.0)	p = 0.859
JB–ME median (IQR) *mm*	7.10 (3.83–9.78)	7.10 (3.83–10.7)	p = 0.915	7.10 (4.30–10.2)	7.01 (3.94–10.5)	p = 0.746
JB–IAC median (IQR) *mm*	7.56 (6.11–10.1)*	6.78 (4.97–8.17)*	p = 0.043	8.14 (6.18–8.85)	7.21 (5.75–8.77)	p = 0.292

*mm* millimeters, *IQR* Interquartile range, *SS* sigmoid sinus, *JB* jugular bulb, *EAC* internal acoustic canal, *ME* middle-ear, *IAC* internal acoustic canal.

*Significant different with p < 0.05.

### Association between the degree of pneumatization and morphometry of SS and JB to ear regions

As presented in Table 4, it was observed that the distances between SS and selected ear regions and between JB and the selected ear regions increase in the different degrees of pneumatization of the TB on both sides, with significant differences at p < 0.05. Hyper-pneumatization was observed to be associated with increased distances for all measurements. Overall, a significant association was observed between TB pneumatization and distances of SS and JB to selected ear regions at p < 0.05 and p < 0.01.

**Table 4:** Association between pneumatization of temporal bone and distances of the sigmoid sinus and jugular bulb to selected ear regions (external acoustic canal, middle-ear & internal acoustic canal).

	Right pneumatization					Left pneumatization				
	Hypo (N = 28)	Moderate (N = 44)	Good (N = 45)	Hyper (N = 65)	p-value	Hypo (N = 28)	Moderate (N = 41)	Good (N = 43)	Hyper (N = 75)	p-value
SS–EAC median (IQR) *mm*	14.6 (11.6–16.7)^a^	15.0 (13.1–16.9)	16.0 (13.8–17.8)	16.4 (14.1–17.6)^a^	0.015*	13.4 (12.8–16.9)^acd^	15.7 (13.9–17.8)^c^	16.0 (14.1–19.2)^d^	16.5 (14.7–18.2)^a^	0.007**
SS–ME median (IQR) *mm*	12.3 (9.85–13.4)	12.6 (10.4–15.1)	12.9 (10.2–14.9)	13.5 (11.8–15.0)	0.017*	11.5 (10.1–13.5)^a^	12.8 (11.2–13.9)	12.8 (11.7–14.4)	13.6 (11.5–14.9)^a^	0.021*
SS–IAC median (IQR) *mm*	18.0 (15.9–21.0)	19.3 (16.4–22.3)	19.5 (16.2–21.4)	19.5 (16.5–22.1)	0.019**	19.8 (16.8–21.1)^ad^	19.8 (17.2–20.1)^be^	20.2 (19.2–22.1)^de^	20.3 (19.2–22.8)^ab^	0.003**
JB–EAC median (IQR) *mm*	10.9 (7.97–15.2)^a^	13.1 (10.2–15.4)	13.7 (10.5–16.3)	14.0 (10.2–15.4)^a^	0.011*	10.0 (8.34–14.0)^a^	12.9 (11.0–15.2)	13.9 (9.72–16.7)	14.0 (11.4–16.4)^a^	0.002**
JB–ME median (IQR) *mm*	5.07 (2.63–7.15)^a^	7.18 (4.11–11.6)	7.62 (4.53–10.5)	8.40 (6.68–10.3)^a^	0.008**	4.85 (2.85–7.70)^a^	6.61 (4.42–12.5)	7.85 (4.05–9.15)	8.69 (5.15–10.5)^a^	0.012*
JB–IAC median (IQR) *mm*	6.50 (5.02–7.68)	6.98 (6.01–8.01)^b^	7.11 (5.65–10.4)	7.68 (6.13–10.4)^b^	0.011*	6.70 (5.70–8.68)^a^	7.31 (5.51–8.85)	7.32 (5.78–8.68)	8.14 (6.55–8.85)^a^	0.046*

*mm* millimeters, *IQR* Interquartile range, *SS* sigmoid sinus, *JB* jugular bulb, *EAC* internal acoustic canal, *ME* middle-ear, *IAC* internal acoustic canal.

*Significant association* at *p < 0.05, **p < 0.01 with *significant difference,* a, b, c, d, e, (Kruskal–Wallis test followed by Wilcoxon Signed Rank test).

^a^hypo vs. hyper.

^b^good vs. hypo.

^c^hypo vs. moderate.

^d^hypo vs. good.

^e^moderate vs. good.

### Variations in morphology of vasculature

The incidence of anatomical variations and morphologies of the sigmoid sinus, jugular bulb, and internal carotid artery in the selected study population is summarized in Table 5. All vascular variants included in this study were seen to be present regardless of the age of the patients or their population groups in the study population.

**Table 5:** Incidence of anatomical variations of the sigmoid sinus, jugular bulb, and internal carotid artery.

Vascular Variations	Female				Male				Overall			
	Left *f (%)*	Right *f (%)*	p-value	Total *f (%)*	Left *f (%)*	Right *f (%)*	p-value	Total *f (%)*	Left *f (%)*	Right *f (%)*	p-value	Total *f (%)*
SS			0.650				0.148				0.119	
Half-moon	5 (5.6)	3 (3.4)		8 (4.5)	3 (3.1)	4 (4.6)		7 (3.8)	8 (4.3)	7 (4.0)		15 (4.2)
Protrusive	34 (38.2)	38 (43.7)		72 (40.9)	23 (23.7)	31 (35.6)		54 (29.3)	57 (30.6)	69 (39.7)		126 (35.0)
Saucer	50 (56.2)	46 (52.9)		96 (54.5)	71 (73.2)	52 (59.8)		123 (66.8)	121 (65.1)	98 (56.3)		219 (60.8)
JB			0.289				0.518				0.189	
Flat JB	5 (5.7)	5 (5.8)		10 (5.7)	7 (7.3)	8 (8.9)		15 (8.1)	12 (6.5)	13 (7.3)		25 (6.9)
High JB	30 (34.5)	32 (36.4)		62 (35.4)	23 (24.0)	30 (33.2)		52 (28.0)	55 (29.9)	60 (33.8)		115 (31.85)
JB dehiscence	0 (0.0)	4 (4.6)		4 (2.3)	1 (1.0)	2 (2.2)		3 (1.6)	1 (0.5)	6 (3.4)		7 (1.9)
ICA			0.041*				0.040*				0.030*	
Dehiscence	2 (2.3)	4 (4.5)		6 (3.4)	1 (1.0)	6 (6.6)		7 (3.7)	3 (1.6)	10 (5.6)		13 (3.5)

X^2^, significant different at *p < 0.05.

*f* frequency, *%* percentage, *SS* sigmoid sinus, *JB* jugular bulb, *ICA* internal carotid artery.

### Sigmoid sinus (SS)

Overall, the saucer-shaped and protrusive types of SS were more common, with the former having a higher incidence. Saucer-shaped SS was observed to have an incidence of 219 (60.9%) with a non-significant (X^2^, p = 0.199) dominance on the left TB (121, 65.1%). Protrusive SS was observed to have an incidence of 126 (35.0%) with a non-significant (X^2^, p = 0.199) dominance on the right TB (69, 39.7%). The half-moon type of SS was observed to have the lowest incidence (4.2%). For sex, the saucer-shaped SS was observed to be higher in males with an incidence of 123 (66.8%) and non-significant dominancy on the left for both sexes (male left- 71 (73.2%), Fisher’s, p = 0.148; female left- 50 (56.2%), Fisher’s, p = 0.659). While, the protrusive SS was observed to be higher in females with an incidence of 72 (40.9%) and dominancy on the right for both sexes (female right- 38 (43.7%), Fisher’s, p = 0.659; male right- 31 (35.6%), Fisher’s, p = 0.148).

### Jugular bulb

Jugular bulb variants observed in the study were FJB- 25 (6.9%) (Fig. 2a), HJB- 115 (31.8%) (Fig. 2b), and JB Dehiscence- 7 (1.9%) (Fig. 2c) in which the right TB (right ear) tends to have a higher incidence of 13 (7.3%), 60 (33.8%) and 6 (3.4%), respectively. Non-significant side dominancy was also observed in relation to sex (female- Fisher’s, p = 0.289; male- Fisher’s, p = 0.518), with the female having more incidence of HJB (62 (35.4%)) and JB Dehiscence (4 (2.3%)), while the male showed more incidence of FJB (15 (8.1%)).

### Internal carotid artery

ICA dehiscence incidence was observed to be 13 (3.5%), with males having an incidence of 7 (3.7%) and females having an incidence of 6 (3.4%). The incidence was also more dominant in the right ear (right TB) in the overall incidence and with sex at p < 0.05.

### Association between degrees of pneumatization and vascular variants

For both right and left, vascular variants in the shape of SS analyzed in this study were observed to occur in the different degrees of pneumatization, with the most occurrence of half-moon-shaped SS in patients with hypo-pneumatized TB. In contrast, protrusive and saucer-shaped SS occurs more in hyper-pneumatized patients. However, there was no significant association between the incidences of these variable shapes of SS and the degree of TB pneumatization (Rt- p = 0.070; Lt- p = 0.645).

For the analyzed JB variants in the study, HJB was observed in the different degrees of pneumatization but with a high incidence in patients having hyper-pneumatized TB on both sides (Rt- 63.3%; Lt- 51.4%) with a significant association at p < 0.001. JB dehiscence was also observed to have a significant association (p < 0.001) with hyper-pneumatization on both sides, mostly occurring in patients with HJB. However, more incidence of FJB was observed in patients with hypo-pneumatized TB with a significant association at p < 0.001.

Lastly, the incidence of ICA dehiscence was observed only in patients with hyper-pneumatized TB on both sides, showing a significant association of p < 0.01. The results are detailed in Table6.

**Table 6:** Association between pneumatization of the temporal bone and some selected vascular variants of the sigmoid sinus, jugular bulb, and internal carotid artery.

	Right temporal bone pneumatization					Left Temporal bone pneumatization				
	Hypo (N = 28)	Moderate (N = 44)	Good (N = 45)	Hyper (N = 65)	p-value	Hypo (N = 28)	Moderate (N = 44)	Good (N = 45)	Hyper (N = 65)	p-value
SS					0.070 (NSA)					0.645 (NSA)
Half-moon	4 (14.8%)	2 (4.5%)	1 (2.3%)	0 (0.0%)		3 (11.1%)	2 (4.9%)	1 (2.3%)	2 (2.7%)	
Protrusive	7 (25.9%)	19 (43.2%)	16 (37.2%)	27 (45.0%)		9 (33.3%)	13 (31.7%)	13 (30.2%)	21 (28.4%)	
Saucer	16 (59.3%)	23 (52.3%)	26 (60.5%)	33 (55.0%)		15 (55.6%)	26 (63.4%)	29 (67.4%)	51 (68.9%)	
JB					< 0.001**					< 0.001**
Flat JB	5 (18.5%)	4 (6.8%)	3 (6.7%)	1 (2.2%)		5 (10.7%)	4 (9.8%)	3 (0.0%)	0 (6.9%)	
High JB	2 (7.4%)	9 (20.0%)	10 (22.7%)	38 (63.3%)		6 (21.4%)	5 (12.2%)	7 (16.3%)	37 (51.4%)	
JB dehiscence	0 (0.0%)	0 (0.0%)	2 (4.5%)	4 (6.7%)		0 (0.0%)	0 (0.0%)	0 (0.0%)	1 (1.4%)	
ICA dehiscence					0.002*					0.009*
Yes	0 (0.0%)	0 (0.0%)	0 (0.0%)	10 (16.8%)		0 (0.0%)	0 (0.0%)	0 (0.0%)	3 (4.0%)	

Fisher’s test showing significant association at *p < 0.01, **p < 0.001.

*NSA* no significant association, *SS* sigmoid sinus, *JB* jugular bulb, *ICA* internal carotid artery.

## Discussion

The degree of TB pneumatization has been implicated as one of the factors influencing the relative morphology and location of its related blood vessels in relation to the ear regions^2,11^. However, observable and measurable data relating this to different degrees of pneumatization of TB remains scarce. Moreso, previous studies have highlighted that there is still a paucity of data to ascertain the association between different degrees of pneumatization of the TB anatomical variations of its related vessels and their relationship with ear regions Dai et al*.*^26^ and Singh et al*.*^9^.

In this study, the classification of Han et al*.*^25^ was utilized by evaluating air cells around the sigmoid sinus and classifying the degrees of TB pneumatization into hypo-, moderate-, good-, and hyper-pneumatization. All degrees of pneumatization existed in the present study population regardless of age or population group. Overall, hyper-pneumatization had the highest incidence (37.9%), and hypo-pneumatization had the lowest incidence (15.2%), which is the same as reported by Tan et al*.*^27^ in the Singapore population. Bronoosh et al*.*^28^ also identified hyper-pneumatization as the most common pattern occurring in 30.9% of their study subjects in an Iranian population.

Generally, the position and location of TB-related vasculature to ear regions vary greatly among individuals and between right and left ears which corresponds to the findings from the present study^26,29–35^. No significant difference was observed with age or among population groups. However, the present study identified the distances between SS and JB and the selected ear regions, i.e., EAC, ME, and IAC, to vary significantly between right and left, with the left significantly higher than the right for all distances (p < 0.05, p < 0.01, p < 0.005).

All distances measured were highest in patients with hyper-pneumatized TB. Significant associations (p < 0.05, p < 0.01) existed between the different degrees of pneumatization and all distances measured. This was because distances between SS and JB and the selected ear regions increase as pneumatization extends across each of the three parallel lines anterolaterally applied at angle 45°, which was utilized to classify the degree of pneumatization in this study. According to Lima et al*.*^36^, mastoid surface areas and volume follow a linear correlation. This implies that an increased pneumatization denotes an increased surface area, which increases the distances observed between these vessels and ear regions as pneumatization increases.

The present study also analyzed the incidence of some variants of vessels such as SS, JB, and ICA relative to TB structures, which have implications for the development of pathologic abnormalities, such as encroachment on the structures of the middle ear or eroding into the inner ear^37^. These, of course, are encountered in routine clinical practice and may pose dangers in ear-related and mastoid surgeries in which surgeons need to avoid such pitfalls^9,11,16,38^. The SS and its variant shapes were observed in the different degrees of pneumatization for right and left. Although in the reviewed literature, the half-moon-shaped SS had been ascribed to a healthy mastoid and was seen as the most common type of SS^39^, the present study identified the SS as more saucer-shaped in the study population with no significant association with the degree of pneumatization. In contrast, half-moon-shaped SS was found more in patients with hypo-pneumatized TB; however, there was no significant association.

For JB and its anatomical variants, HJB was found to be the most common variant of JB. This high incidence conforms with the studies of Aslan et al*.*^4^ and Kawano et al*.*^38^, who reported high incidences of HJB to be 23% and 16%, respectively, and supports the claims of Friedmann et al*.*^8^ and Park et al*.*^40^ that HJB is the most common JB variant not rare in a population. This, however, is significantly associated (p < 0.001) with increasing pneumatization. This implies that, as pneumatization extends the three parallel lines, most significantly where pneumatization extends beyond the arbitrary line drawn at the most posterior point of SS (hyper-pneumatization), increase in the incidence of HJB was observed. Although JB abnormalities are rarely encountered during ear surgeries, a high jugular bulb remains a recognized problem that can lead to brisk venous haemorrhage if the bulb is inadvertently opened^41^. Hence, ENT surgeons should watch out for the possibility of a high-riding jugular bulb in patients with hyper-pneumatized TB.

Like HJB, this study also observed a significant association between hyper-pneumatization and JB dehiscence. The similarity in the significant association observed for HJB and JB dehiscence follows their anatomical relationship. According to Gaillard^42^, HJB becomes a dehiscent JB when the sigmoid plate between high-riding JB and the middle ear is missing, allowing the JB to bulge into the middle ear cavity. This means that an individual with hyper-pneumatized TB is at risk of having a dehiscent JB in the absence of the sigmoid plate, which is one of the common causes of pulsatile tinnitus^43^.

In addition, a lower incidence of flat JB was observed in this study. This contradicts the report of Vachata et al*.*^16^, who documented a higher incidence of FJB. A significant association between decreasing pneumatization and incidence of FJB was observed, especially when pneumatization is limited to the first anteromedial line, i.e., hypo-pneumatization or nothing near this line. In this case, the mastoid contains more marrow than air cells (diploic) or contains mainly dense bone^44,45^, which could inhibit the formation of the bulb.

Furthermore, ICA dehiscence as a vascular variant was considered in this study. The dehiscence of ICA was observed to occur with an incidence of 3.5% from the analyzed scans, which was only seen in the TB to be significantly associated with hyper-pneumatized TBs with extensive pneumatization. An extensive pneumatization is when pneumatization spreads to the petrous apex (and even beyond), where the ICA is seen to exit the carotid canal into the foramen lacerum to enter the skull^1,46^. The transition of the bony petrous into air cells in an extensive pneumatized could result in dehiscence of the ICA due to the loss of bony elements.

Importantly, in ENT and its related surgeries, most of these vascular variants can result in ear disturbances and dysfunction, as well as diagnostic and surgical difficulties^47^. Although middle ear disease such as chronic otitis media is one common cause of hearing loss, high jugular bulb (HJB), for instance, when associated with diverticulum or dehiscence high-riding JB, has been reported to result in sensorineural hearing loss, conducting hearing loss, and Meniere-like syndrome (a disorder of inner ear that can lead to dizzy spell and hearing loss common to the left ear) due to compression of the inner ear^47–52^. The high incidence of HJB and JB dehiscence reported in this study due to increased pneumatization (hyper-pneumatization- 37.9%) could also contribute to hearing loss in children, teenagers, and adults.

## Conclusion

This study investigated the association between the degree of pneumatization and TB-related vasculature in terms of their morphologies and morphometric relationship with ear regions/structures. Hyper-pneumatization was more common in the study population and significantly associated with HJB, JB dehiscence, and ICA dehiscence, while hypo-pneumatization was least common and significantly associated with FJB. No significant association between the degree of pneumatization and SS variants. Furthermore, distances of SS and JB to ear regions increase as the degree of pneumatization increases. Hence, this study concludes that TB pneumatization is a factor that influences the relative morphological variants of JB and ICA only and the distances of SS and JB to different ear regions. Awareness of these will contribute significantly to predicting and avoiding relative morphological variants and the location of these vessels, which are recognized pitfalls that could become distressful or cause complications during ear-related surgeries.

## Data Availability

Data are available upon reasonable request and with permission from the KwaZulu-Natal Department of Health, South Africa.

## Abbreviations

EAC: External acoustic canal
IAC: Internal acoustic canal
ICA: Internal carotid artery
JB: Jugular bulb
LSCC: Lateral semicircular canal
ME: Middle ear
TM: Tympanic membrane
TB: Temporal bone
SS: Sigmoid sinus
